# A snapshot love story: what serial crystallography has done and will do for us

**DOI:** 10.1107/S2059798324005588

**Published:** 2024-07-10

**Authors:** Alessandra Henkel, Dominik Oberthür

**Affiliations:** a Center for Free-Electron Laser Science CFEL Deutsches Elektronen-Synchrotron DESY Notkestr. 85 22607Hamburg Germany; University of Oxford, United Kingdom

**Keywords:** serial crystallography, time-resolved, structural dynamics

## Abstract

Serial crystallography, born from groundbreaking experiments at the Linac Coherent Light Source in 2009, has evolved into a pivotal technique in structural biology. Initially pioneered at X-ray free-electron laser facilities, it has now expanded to synchrotron-radiation facilities globally, with dedicated experimental stations enhancing its accessibility. This review gives an overview of current developments in serial crystallography, emphasizing recent results in time-resolved crystallography, and discussing challenges and shortcomings.

## Abstract 2

This article gives an overview of current trends and challenges in serial crystallography, with a focus on results from time-resolved experiments.

## 1. Introduction

In 2009, a large collaboration of scientists gathered at the experimental station for ‘atomic, molecular and optical physics’ or AMO at the Linac Coherent Light Source (LCLS), Stanford, USA (Emma *et al.*, 2010 ▸) entered so far uncharted territory: shooting with femtosecond short soft X-ray pulses from an X-ray free-electron laser (XFEL) at very small protein crystals randomly dispensed in a liquid jet of a few micrometres in thickness, recording the resulting diffraction patterns and trying to make sense of what was recorded. A few years earlier some of the scientists involved in this experiment had already shown that similar pulses generated at the FLASH XUV free-electron laser at DESY, Hamburg, Germany could be used to record diffraction patterns before the destruction of the sample (Chapman *et al.*, 2006 ▸) by the very same pulses and that the recorded diffraction could be used to reconstruct the 2D shape of a silicon nitride membrane. In 2009, however, it was unclear if what was successful for the 2D reconstruction of a macroscopic object would work in 3D and for a sample with submicroscopic features. Each protein crystal would only be probed once, and within the pulse of a few femtoseconds in duration the movement of the crystal would be negligible. If the overlap between the jet and the pulsed X-ray beam allowed the recording of crystal diffraction, the resulting diffraction images would be ‘still’ snapshots with no orientational relation to the image recorded before or after. This also meant that the existing software for crystallo­graphic data processing, which relied on information on the relation of crystal orientation between recorded images, could not be used.

**Figure 1:**
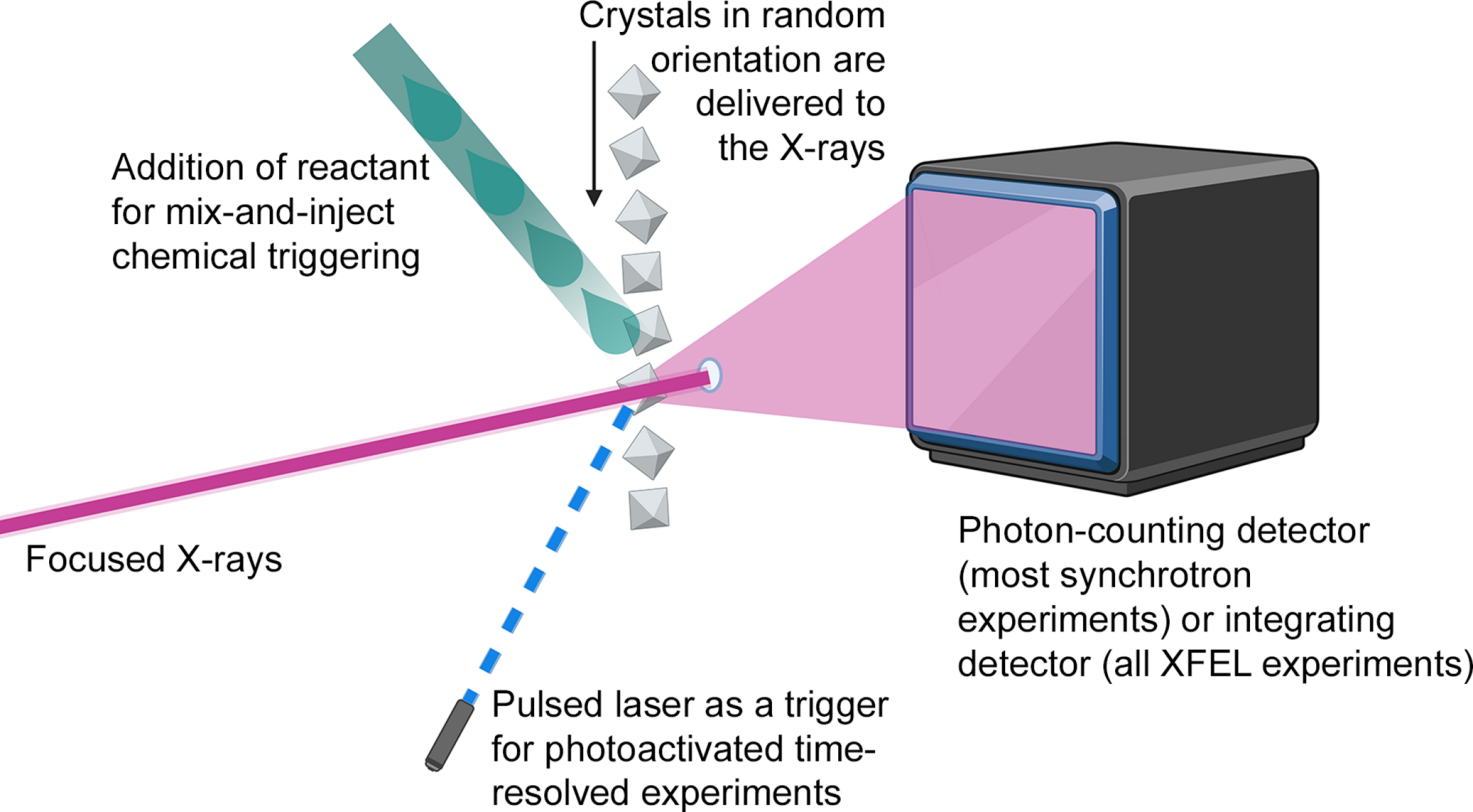
Diagram of a serial crystallography experiment using either an X-ray free-electron laser (XFEL) or synchrotron radiation. Protein crystals are delivered to the X-ray beam in random orientations. The intense X-rays interact with the crystals, producing diffraction patterns that are recorded by a detector. Every crystal is only probed once. Time-resolved experiments can be conducted either by light activation using a laser or by chemical activation. In the latter case the reactant is mixed with the protein crystals at a defined time delay before probing the crystals with X-rays. (Created with BioRender.com.)

The large collaboration was ultimately successful (Chapman *et al.*, 2011 ▸) and what was born in 2009 is now known as serial crystallography (SX; Fig. 1 ▸), a term introduced by John C. H. Spence and Bruce R. Doak in 2004 to describe something different: a method to continuously deliver single hydrated protein particles in liquid helium droplets to an electron beam to record single-molecule electron diffraction data (Spence & Doak, 2004 ▸). This never materialized, but the idea of continuous delivery of sample was realized using protein nanocrystals to record powder diffraction patterns at the Advanced Light Source (ALS) in Berkeley (Shapiro *et al.*, 2008 ▸) and then in 2009 in the first ‘modern’ SX experiments (Chapman *et al.*, 2011 ▸). Since then, techniques to bring the protein crystals into the X-ray beam have been developed further (Sierra *et al.*, 2018 ▸; Grünbein & Nass Kovacs, 2019 ▸). To analyze the data from SX experiments, specialized software has been developed (White *et al.*, 2016 ▸; Hattne *et al.*, 2014 ▸). About 15 years after the groundbreaking experiments at AMO, SX (Schlichting, 2015 ▸; Barends *et al.*, 2022 ▸) has become an important part of structural biology and offers an additional approach for the investigation of macromolecular structures, especially with regard to the investigation of time-resolved protein dynamics (Barends *et al.*, 2015 ▸, 2022 ▸; Fadini *et al.*, 2023 ▸; Christou *et al.*, 2023 ▸; Stagno *et al.*, 2017 ▸; Coquelle *et al.*, 2018 ▸; Pande *et al.*, 2016 ▸; Brändén & Neutze, 2021 ▸). SX nowadays can not only be conducted at the five existing XFEL facilities as serial femtosecond crystallography (SFX), but also at many synchrotron-radiation facilities around the globe, which is then termed serial synchrotron crystallography (SSX). The experimental stations T-REXX (PETRA III, EMBL, Germany), ID29 (ESRF, France) and MicroMAX (MAX IV, Sweden) were even built as facilities focused on SX (Caramello & Royant, 2024 ▸).

There are four general advantages of SX. (i) Since every crystal is only probed by X-rays once there is generally less accumulated radiation damage, as each diffraction image results from a fresh rather than a previously shot and damaged crystal (Garman & Weik, 2023 ▸). At XFELs, diffraction data can be collected using pulses that are typically only 20–40 fs long or even shorter. At such exposure times data collection is almost radiation damage-free at dose levels orders of magnitude higher (Chapman *et al.*, 2014 ▸) than the accepted limits for room-temperature data collection of about 400 kGy (de la Mora *et al.*, 2020 ▸; Southworth-Davies *et al.*, 2007 ▸). The extent of radiation damage in data collected at XFELs is still under discussion (Nass, 2019 ▸; Nass, Gorel *et al.*, 2020 ▸; Chapman *et al.*, 2014 ▸), although in general it can be said that shorter XFEL pulses should result in less radiation damage (Nass, 2019 ▸). This is in line with earlier simulations for single-particle diffractive imaging (Neutze *et al.*, 2000 ▸), with the difference that slightly longer pulses can be tolerated in crystallography (Barty *et al.*, 2012 ▸). (ii) As a result of the lower rate of radiation damage and the fact that each crystal is probed only once, data collection under noncryogenic conditions is possible without special precautions. This enables data collection at room temperature and even physiological temperatures, with strong positive implications for the investigation of protein structural dynamics. (iii) Much smaller crystals can be used. At synchrotrons, approximately as small as the focal spot of the X-rays at maximum flux, and at XFELs, currently as small as about 200–300 nm (Gati *et al.*, 2017 ▸; Williamson *et al.*, 2023 ▸). (iv) SX gives access to time-resolved studies of irreversible dynamics in biological macromolecules and in general facilitates time-resolved crystallography (Caramello & Royant, 2024 ▸). Moreover, time-resolved SFX (trSFX) enabled the study of ultrafast sub­pico­second processes in biological systems with crystallo­graphy for the first time (Barends *et al.*, 2015 ▸; Pande *et al.*, 2016 ▸), a decrease of three orders of magnitude in time compared with what was possible before (Schotte *et al.*, 2012 ▸).

In this review, we provide a brief overview of current trends in SX, with a focus on developments for, and the results of, time-resolved crystallography and the challenges along the way. It was inspired by the micro-symposium *Room-temperature Serial Snapshot Micro-crystallography: Highlights from XFELs and Synchrotrons* at the 26th Congress and General Assembly of the International Union of Crystallography (IUCr) in Melbourne, which we chaired.

## 2. Current trends in serial and time-resolved crystallography

### 2.1. Structure determination

At the time of the first successful SX experiments, the new method was met with enthusiasm with regard to its possible role in the elucidation of structures of biological macromolecules. It was thought that the changed requirements in crystal size and reduced radiation damage would enable successful data collection on systems that were out of the reach of traditional crystallography. Serial femtosecond crystallography (SFX) delivered remarkable results, for example for *in vivo* crystallized proteins (Redecke *et al.*, 2013 ▸; Nass, Redecke *et al.*, 2020 ▸; Duszenko *et al.*, 2015 ▸) and allowed some GPCR structures to be elucidated for the first time, such as the rhodopsin–arrestin complex (Kang *et al.*, 2015 ▸). In parallel, impressive developments in cryo-transmission electron microscopy (cryoEM) enabled structure elucidation of large macromolecular complexes in unprecedented detail (Kühlbrandt, 2014 ▸). The wider availability of state-of-the-art electron microscopes and easier access compared with beamtime at XFELs, combined with the comparatively reduced amount of sample (see Section 3) required for data collection using cryoEM, as well as the removal of the crystallization step, has shifted the focus away from using SFX for such structure-elucidation purposes. Structures of GPCRs, without the mutations and modifications necessary for crystallography, can now regularly be solved using cryoEM (García-Nafría & Tate, 2021 ▸).

There are, however, targets that need to be studied in crystalline form because their crystalline state is of biological importance. Many proteins that natively crystallize *in vivo* fall into this category. Some bacteria, such as *Bacillus thuringiensis*, produce toxins that target specific insects. These bacterial insecticides are being widely used in agriculture, in genetically modified plants and also tentatively to combat vectors of infectious diseases. These insecticides are proteins, are produced during sporulation and are stored as native nanocrystals. The crystals are ingested by insects, dissolve in the digestive tract, are activated by proteases and, if in the target organism, bind to specific receptors on the gut wall and finally kill the insect. The crystals are too small for synchrotron crystallography and recrystallization is often only possible with heavy mutations or not at all. SFX was used to solve the structures of such insecticides at room temperature (Colletier *et al.*, 2016 ▸; Williamson *et al.*, 2023 ▸; Tetreau *et al.*, 2020 ▸, 2022 ▸) at resolutions of up to 1.62 Å (Williamson *et al.*, 2023 ▸). Recently, a team at SPring-8 were able to solve the structure of a native insecticide using a synchrotron light source (Tanaka *et al.*, 2023 ▸), albeit at much lower resolution and under cryogenic conditions. Similarly, the structure of nudivirus occlusion body protein was determined from native crystals at Diamond Light Source (Keown *et al.*, 2023 ▸). The resolution achieved (1.7 Å) was comparable to the resolution reported for the Tpp49Aa structure determined at EuXFEL, but the nudivirus crystals were much larger (2–5 µm) than the insecticide crystals used at EuXFEL (about 500 nm). In the future, electron crystallography might be used for structure determination of such insecticides from native nanocrystals; however, at the cost of having to collect data under cryogenic conditions rather than at room temperature.

The advent of artificial intelligence-based 3D structure prediction of biological macromolecules (Jumper *et al.*, 2021 ▸) and their interactions with each other and with potential small-molecule binders (Krishna *et al.*, 2024 ▸) was, and continues to be, the next revolution in structural biology after the ‘resolution revolution’ in cryoEM. Its implications for SFX, however, are different. On one hand, *AlphaFold* and similar tools drastically expanded the search-model space for molecular replacement, which means that experimental phasing is now only needed for very few targets, if at all. On the other hand, one of the shortcomings of these prediction models is that they have been trained on the Protein Data Bank (PDB; Berman *et al.*, 2003 ▸). The majority of the protein models in the PDB were built using data collected under cryogenic conditions (usually around 100 K), and furthermore for many proteins only a single state of the protein was modeled and deposited in the PDB. The data set used to train the prediction models is biased towards a cryogenic ground state that is far away from physiological pH and temperature conditions. Here, SFX can play a huge role in the future by delivering accurate structures of biological macromolecules in bound and unbound states, under aerobic and anaerobic conditions and collected at physiological pH and temperature.

### 2.2. Time-resolved structural studies

**Figure 2:**
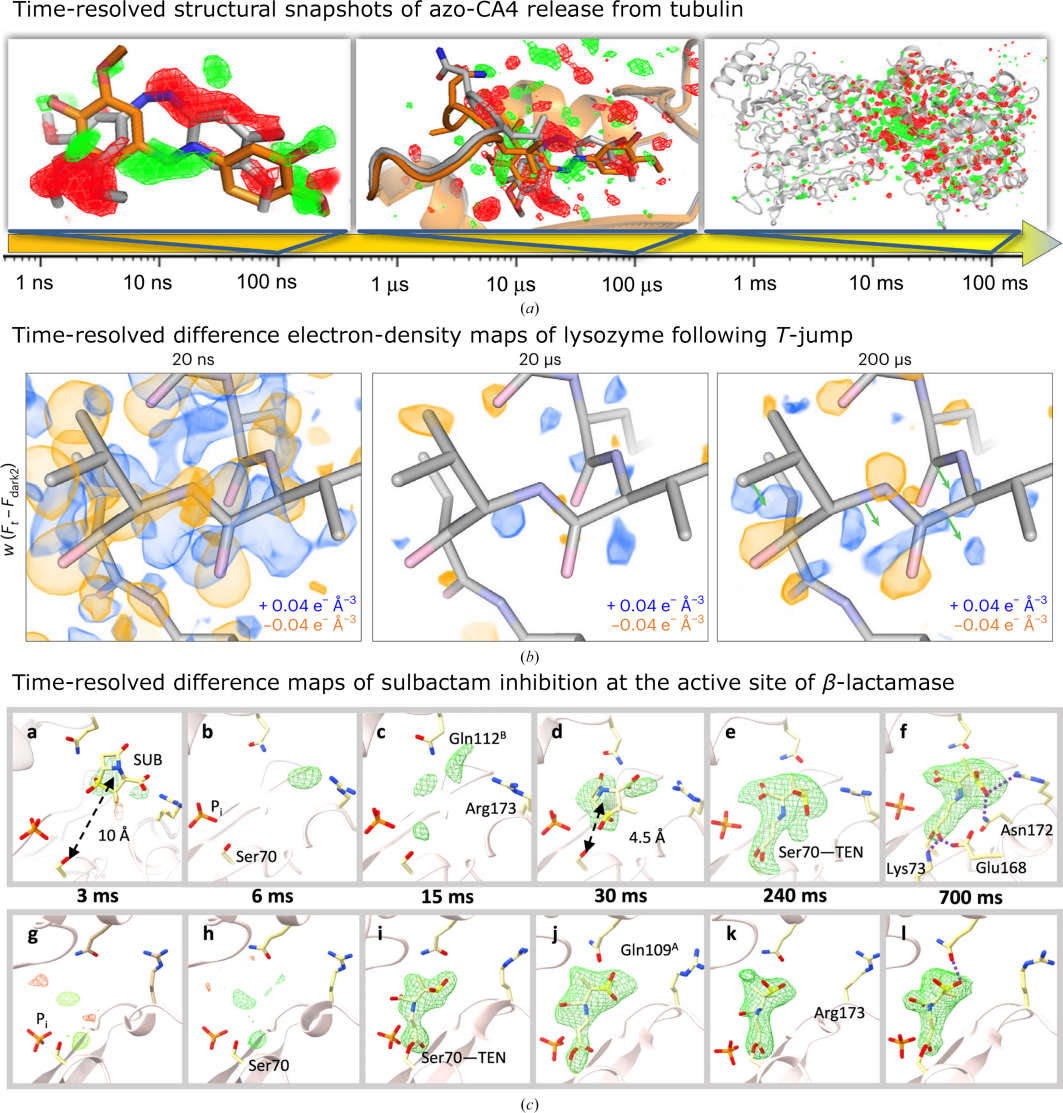
Examples of results from time-resolved SX using three different methods for reaction initiation. (*a*) Time-resolved structural snapshots of the release of azo-CA4 from tubulin (Wranik, Weinert *et al.*, 2023 ▸). The time arrow depicts the investigated time regime. The panels from left to right show the isomorphous difference maps obtained at 100 ns with changes centered on the ligand, 100 µs with changes centered on the binding pocket and 100 ms with conformational changes propagating throughout the protein. All panels show isomorphous difference maps in red (negative) and green (positive) at 3σ. The structure in the given time range (colored orange) is compared with that in the previous time range (colored gray). (*b*) shows the time-resolved difference electron-density (DED) maps of lysozyme following a temperature jump (*T*-jump; Wolff *et al.*, 2023 ▸). Weighted DED maps are depicted for each time point around residues 97–100 of lysozyme visualized at an absolute contour level of ±0.04 e^−^ Å^−3^. Positive DED is displayed as a blue map and the negative DED map is shown in orange. The green arrows depicted at the 200 µs time step indicate proposed coordinate motions derived from analysis of the DED. (*c*) Difference electron-density maps around the active center of both subunits (subunit A, top row; subunit B, lower row) of a lactamase at different time points of a mix-and-inject time-resolved experiment with the lactamase inhibitor sulbactam (Malla *et al.*, 2023 ▸). Omit maps are shown in all subpanels except for subpanels d, f, j and l, which show polder maps (contour levels ±3σ). SUB, sulbactam; TEN, *trans*-enamine. Images and captions were taken and adapted from (*a*) Wranik, Weinert *et al.* (2023 ▸), (*b*) Wolff *et al.* (2023 ▸) and (*c*) Malla *et al.* (2023 ▸), licensed under a Creative Commons Attribution 4.0 International License (https://creativecommons.org/licenses/by/4.0/).

The unique selling point of SX to date is the ability to study protein dynamics by time-resolved methods (Fig. 2 ▸). Time-resolved methods were originally introduced in the late 1980s, when the development of synchrotron-radiation sources, detectors and software allowed them (Brändén & Neutze, 2021 ▸). As outlined in Section 1, SX is very well suited for time-resolved methods. Depending on the pulse lengths of both the pump and the probe, time resolutions of a few hundred femto­seconds are currently achievable for dynamics that can be induced by photons (using femtosecond pulsed laser sources) and probed by XFELs (with pulse lengths as short as a few hundred attoseconds). There are challenges in such experiments that will be discussed later in this text, but overall this has been a remarkable success story, starting from the seminal studies on photoactive yellow protein (PYP; Tenboer *et al.*, 2014 ▸; Pande *et al.*, 2016 ▸) and myoglobin (Barends *et al.*, 2015 ▸), and continuing with studies on DNA repair (Maestre-Reyna *et al.*, 2023 ▸; Christou *et al.*, 2023 ▸) and photosynthesis (Young *et al.*, 2016 ▸; Suga *et al.*, 2019 ▸; Bhowmick *et al.*, 2023 ▸; Kern *et al.*, 2018 ▸).

#### 2.2.1. Direct photoactivation

In general, photoactivation in proteins requires a chromophore in which the absorption of a photon results in the excitation of electronic states. The excitation is followed by a specific decay of these states through various pathways, which include chemical modification of the chromophore (such as *cis*–*trans* or *trans*–*cis* isomerization) through photochemistry, electron transfer through the protein via various cofactors and/or dynamic response of the protein. These pathways result in the catalysis of chemical reactions (DNA repair in photolyases or water splitting in photosystem II) or signal transduction (as in PYP or rhodopsin). What all chromophores have in common is an extended system of delocalized π-electrons. In past years, chromophores such as flavin adenine dinucleotide (FAD), heme and retinal have been prime targets for time-resolved approaches.

Retinal is the chromophore present in most photoreceptors and its interaction with light is the first step in a signal transduction cascade that enables vision in humans and animals. Recently, ultra-fast processes occurring in bovine rhodopsin upon the excitation of retinal were investigated via trSFX (Gruhl *et al.*, 2023 ▸). Another protein with retinal as a chromophore is bacteriorhodopsin, in which it does not confer vision but is used for energy conversion to generate chemical energy for the organism. The dynamics of bacteriorhodopsin have been elucidated using both SFX and SSX, spanning approximately 12 orders of magnitude in time from femto­seconds to seconds (Nango *et al.*, 2016 ▸; Nogly *et al.*, 2018 ▸; Weinert *et al.*, 2019 ▸; Nass Kovacs *et al.*, 2019 ▸). Also, other microbial rhodopsins, which act as ion transporters upon light-driven isomerization of retinal, have been studied extensively by time-resolved crystallography (Mous *et al.*, 2022 ▸; Skopintsev *et al.*, 2020 ▸; Oda *et al.*, 2021 ▸; Yun *et al.*, 2021 ▸).

Heme is a cofactor involved in various biological processes, primarily serving as a prosthetic group in hemoglobin and myoglobin for oxygen transport and storage in vertebrates. It also plays a crucial role in catalyzing redox reactions, for example in cytochromes or as a regulatory factor in various enzymes such as catalases or peroxidases. Due to the extended system of delocalized π-electrons present in its porphyrin structure, heme is photoactive. Carbon monoxide bound to heme in hemoglobin or myoglobin is released upon exposure to light. This photodissociation of carbon monoxide or O_2_ from heme has been studied extensively by various methods over the past 130 years (Alpert *et al.*, 1974 ▸; Austin *et al.*, 1975 ▸; Šrajer *et al.*, 1996 ▸). Both myoglobin and hemoglobin are model systems for studying photo-induced reactions and for developing and critically assessing novel methods, such as trSFX (Barends *et al.*, 2015 ▸). Flash photolysis of carbon monoxide from heme has also been used to study the dynamics of cytochrome *c* oxidase using trSFX (Ishigami *et al.*, 2023 ▸; Safari *et al.*, 2023 ▸).

While retinal and heme were and will always be in fashion, 2023 saw the rise of flavins as chromophores to study with trSFX. Flavins, such as FAD or flavin mononucleotide (FMN), serve as cofactors in a wide range of enzymes, where they participate in electron-transfer reactions, acting as carriers of electrons or H atoms. Most of these proteins are not directly photoactive; however, FAD, in which a riboflavin moiety is attached to adenosine diphosphate, is the chromophore of two photoenzymes: (i) fatty-acid photodecarboxylase and (ii) photolyase. Moreover, FAD is the chromophore in blue light-sensing photoreceptors called BLUFs (blue light sensors using FAD), and similarly FMN acts as the chromophore in LOV (light, oxygen or voltage) domains.

All of these systems have recently been studied by trSFX, starting with an investigation into the photocycle of fatty-acid photodecarboxylase (FAP; Sorigué *et al.*, 2021 ▸). A combination of crystallographic studies with time-resolved spectroscopy and quantum-chemistry calculations showed that light-driven hydrocarbon formation occurred via quasi-instantaneous decarboxylation upon forward electron transfer, coupled with bicarbonate formation involving an active-site water molecule. These findings elucidate a complex mechanism for light-driven hydrocarbon formation by FAP (Sorigué *et al.*, 2021 ▸).

Photolyases can repair DNA damaged by UV photons from, for example, sunlight. There are two distinct photolyases, specialized for either repairing cyclobutane pyrimidine dimers (CPDs) or 6–4 adducts. Since photolyases are photoenzymes with FAD as a chromophore, blue light is required to start the catalytic cycle. In this process FAD is photoreduced to the catalytically active FADH^−^ state (Maestre-Reyna *et al.*, 2022 ▸; Christou *et al.*, 2023 ▸). Two independent teams investigated the DNA-repair mechanism of CPD photolyase from the archaeon *Methanosarcina mazei* (mmCPD) ranging over time scales from picoseconds to microseconds (Maestre-Reyna *et al.*, 2022 ▸, 2023 ▸; Christou *et al.*, 2023 ▸). They observed a bending of FAD as early as 3 ps after photoexcitation and showed that the two bonds of the cyclobutene dimer are cleaved consecutively rather than collectively. A third team investigated ultrafast processes in a DNA-free 6–4 photolyase from *Drosophila melanogaster*. Here, only the response of FAD upon photoexcitation and subsequent electron transfer along a chain of four conserved tryptophan residues was investigated (Cellini *et al.*, 2024 ▸).

Using a combination of cryo-crystallography, cryo-trapping crystallography, FTIR spectroscopy and trSFX, the response of a BLUF domain to photoactivation was investigated. The BLUF domain belonged to a photoactivatable adenylate cyclase from *Oscillatoria acuminata* (OaPAC). It could be shown that light-activation led to a change in the conformation of bound ATP, rendering it energetically favorable for enzyme-catalyzed conversion to cAMP (Chretien *et al.*, 2024 ▸). The response of the FMN-containing Phot-LOV1 domain from *Chlamydomonas reinhardtii* to photoexcitation was investigated on slower time scales (2.5–92.5 ms) using time-resolved SSX (trSSX) at the Swiss Light Source (Gotthard *et al.*, 2023 ▸).

In the last few years, time-resolved studies on photoswitchable variants of green fluorescent protein (GFP) have been published, starting with an investigation into two excited states of the reversibly photoswitchable fluorescent protein rsEGFP2, where it could be shown that the hydroxybenzylidene imidazolinone chromophore adopts a twisted configuration resembling an intermediate between the *trans* and *cis* isomers in one of its excited states (Coquelle *et al.*, 2018 ▸). In a follow-up study, the authors captured an off-to-on switching intermediate of rsEGFP2 formed 10 ns after photoexcitation. This, together with the first study, confirmed that the transition from the off state to the on state in rsEGFP2 occurs through excited-state isomerization within picoseconds, succeeded by microsecond-scale conformational alterations in the ground state, initiating proton transfer within milliseconds (Woodhouse *et al.*, 2020 ▸). Using a modified chromophore, a different group investigated the pathway of chromophore isomerization in rsEGFP2 and showed that it occurs via the so-called ‘hula-twist mechanism’ (Fadini *et al.*, 2023 ▸). A detailed investigation of the structural dynamics of a novel reversibly photoswitchable fluorescent protein and of the origin of contributions to observable changes in electron density on ultrafast (subpicosecond) time scales was presented by the same group (Hutchison *et al.*, 2023 ▸).

However, since most proteins cannot be triggered directly by photons, other ways have to be found to investigate dynamics using time-resolved crystallography.

#### 2.2.2. Photochemical affinity switches

A different class of reactions that can be triggered by photons is based on photochemical affinity switches, in which a photochemical trigger is introduced into a small-molecule ligand binding to a protein. Upon photoexcitation the ligand conformation is changed and thus is the interaction with the protein. This can be utilized in photopharmacology to enable modulation of biological function with light. Azobenzene groups are often used for this purpose, and in a recent study the release of the anticancer compound azo-combretastatin A4 (azo-CA4), which contains such an azobenzene moiety, from tubulin and the subsequent perturbations within the protein could be followed by trSFX and trSSX (Fig. 2 ▸*a*) on time scales ranging from 1 ns to 100 ms (Wranik, Weinert *et al.*, 2023 ▸).

#### 2.2.3. Temperature jump

Utilizing photons to trigger a dynamic response has many advantages, such as the speed of light at which the trigger acts and the precise control over pumping parameters with modern lasers. If the protein cannot be triggered directly, one way to use photons to obtain a response is to influence the environment of the proteins. Protein function is strongly dependent on the temperature of the system. In general, enzyme catalytic activity doubles every 10 K (Schmidt *et al.*, 2010 ▸; Cornish-Bowden, 1979 ▸) up to the limiting temperature at which the protein has maximum activity. By using short laser pulses in the mid-infrared range, water around the protein is heated up rapidly and thus the environment of the protein in a crystal within this water is changed drastically within a few picoseconds. Heat transfer from the solvent through the protein then takes place within a few nanoseconds. The dynamic response of the protein to the temperature jump can be recorded at different time points after the pump, and the associated structural changes can be analyzed. Temperature jumps have been used for time-resolved spectroscopic studies of enzymes for many decades (Hammes, 2008 ▸) and were pioneered by Manfred Eigen in the 1960s. More recently, this was coupled with trSFX to investigate the response of the protein lysozyme, with and without competitive inhibitor bound, to a temperature jump of 21 K (Wolff *et al.*, 2023 ▸; Fig. 2 ▸*b*).

#### 2.2.4. Caged compounds

Even though triggering of protein dynamics by photons has the advantage of a fast and homogeneous trigger, this is simply not possible for most enzymatic reactions. One way to circumvent this is the use of caged compounds, meaning that a chromophore is introduced into the native substrate of an enzyme which is cleaved off by photochemistry, ideally releasing the substrate close to the catalytic center of the enzyme and subsequentially initiating the reaction. Alternatively, the protein is kept at a pH value at which it is inactive in the presence of the substrate, and caged compounds are used that shift the pH of the system upon photoactivation such that the protein is activated. Caged compounds have successfully been used for trSSX. In one case, using a fixed-target platform for sample delivery, hydrolysis of the C—F bond of fluoroacetate by fluoroacetate dehalogenase was studied after release of the substrate from its caged form by photolysis (Mehrabi, Schulz, Dsouza *et al.*, 2019 ▸). In another case, the dependence of enzyme catalysis by an L1 metallo-β-lactamase on the presence of Zn^2+^ ions was exploited. Instead of the substrate, the Zn^2+^ ions required for reaction were released from a caged form through photolysis (Wilamowski *et al.*, 2022 ▸).

#### 2.2.5. Mix-and-inject

The reaction can also be initiated through chemical triggering (Fig. 1 ▸). That is, the protein in its apo state is brought into contact with substrate in a uniform manner to ensure a defined starting point. The substrate is then processed by the enzyme under observation and this process is probed at various time points by time-resolved crystallo­graphy. This method, which is also known as mix-and-inject or mix-and-diffuse, has been utilized at both XFELs and synchrotrons. The fastest achievable time points are very modest in comparison to those possible with photoactivation. However, many enzymatic reactions occur within milliseconds to seconds and the fastest reported mix-and-inject studies were able to achieve mixing times in the single-digit millisecond range (Pandey *et al.*, 2021 ▸; Malla *et al.*, 2023 ▸).

The first successful mix-and-inject experiments used simple mixers upstream of micro-nozzles for jet-based SFX at LCLS to study the binding of the antibiotic ceftriaxone to *Mycobacterium tuberculosis* β-lactamase (Kupitz *et al.*, 2017 ▸) and the reaction states of an adenine riboswitch after exposure to adenine (Stagno *et al.*, 2017 ▸). Improvements in nozzle design (Calvey *et al.*, 2016 ▸, 2019 ▸) for fast and reliable mixing enabled time-resolved studies of *M. tuberculosis* β-lactamase with time delays as short as 30 ms (Olmos *et al.*, 2018 ▸) and subsequently down to 5 ms (Pandey *et al.*, 2021 ▸) and 3 ms (Malla *et al.*, 2023 ▸) (Fig. 2 ▸*c*). Other designs for mixing devices for fast mix-and-inject SFX based on nanoprecision 3D printing have been published (Knoška *et al.*, 2020 ▸; Vakili *et al.*, 2022 ▸), including one approach which enables mix-and-diffuse approaches for high-viscosity sample injection, such as lipidic cubic phase (Vakili *et al.*, 2023 ▸). Using a concentric flow microfluidic electrokinetic sample holder (co-MESH) in mix-and-inject mode, the binding of initiation factor 1 to the small 30S ribosomal subunit could be studied roughly 200 ms after mixing (Yapici *et al.*, 2023 ▸). In a different study, using the same sample-delivery system, changes in the conformational ensemble of isocyanide hydratase during catalysis, both as the wild type (Dasgupta *et al.*, 2019 ▸) and as a mutant (Smith *et al.*, 2024 ▸), were investigated. A combination of mix-and-inject trSFX and molecular-dynamics simulations was used to unravel details of conformational changes during enzyme catalysis.

Another approach utilized at XFELs is based on acoustic droplet ejection onto a moving conveyor belt, which transports the droplets, each containing ideally one protein crystal, into the X-ray beam. This was combined with a piezoelectric injector to add compounds, for example substrate, to the droplets, thus triggering a reaction. The delay time is defined by both the length of the path between the addition of the compound and the X-ray interaction point, and the speed of the tape (Butryn *et al.*, 2021 ▸). This system was used to investigate the *in situ* structural dynamics of a high-spin ferric hydroperoxo intermediate in the P450 enzyme CYP121 (Nguyen *et al.*, 2023 ▸).

The slower time scales of mix-and-diffuse methods should render them ideal for SSX experiments. The first successful mix-and-diffuse SSX experiment, using a conveyor belt (called TapeDrive) to transport crystals into the X-ray focus after mixing with compound, was published not long after the first mix-and-inject experiments at XFELs (Beyerlein *et al.*, 2017 ▸). Another approach utilized fixed-target sample delivery in combination with a piezo-driven droplet generator. Droplets of about 75 pl, containing substrate in either water or crystallization buffer, were dispensed onto protein crystals sitting in wells on a fixed-target chip, with the arrival of the droplet in the respective well being set to be the starting point of the time-resolved measurement (Mehrabi, Schulz, Agthe *et al.*, 2019 ▸). In a third approach, a next-generation TapeDrive was used for mix-and-diffuse time-resolved crystallization of a protein just before probing the crystals with X-rays. The shortest time reported between the start of crystallization and successful structure determination was as low as 2 s (Henkel *et al.*, 2023 ▸). So far, apart from two preprints (Betzel *et al.*, 2023 ▸; Mehrabi *et al.*, 2021 ▸), there are as yet no other examples of the use of mix-and-diffuse SX at synchrotrons, possibly because, despite pioneering developments, the method is still in its infancy (see Section 3).

### 2.3. Drug discovery and temperature

In general, protein function depends strongly on environmental factors such as pressure, pH value, ionic strength of the solution, humidity and/or, as mentioned above, temperature. The degree to which protein dynamics change upon alteration of any of these factors differs from protein to protein. Many enzymes retain catalytic activity in the crystalline state, albeit with slightly different kinetic parameters. Other enzymes are even active in organic solvents (Gupta & Roy, 2004 ▸). In order to understand the function and inhibition of proteins, it is important to take the dependence on these environmental factors into account. In crystallography the protein has to be in a crystal; however, ideally a crystallization condition should be selected that yields crystals which do not impair the essential protein dynamics. Cooling protein crystals to 100 K or below was essential for many achievements in structural biology, as well as in structure-based drug discovery. It facilitated data collection from many targets, yielded data that allowed the calculation of electron-density maps with unprecedented detail and helped to significantly reduce the rate of radiation damage (Garman & Schneider, 1997 ▸; Pflugrath, 2015 ▸). However, it has been shown that cryo-cooling affects the structures and dynamics of proteins (Fraser *et al.*, 2011 ▸; Lang *et al.*, 2014 ▸), that it alters the conformational ensemble of proteins (Keedy *et al.*, 2015 ▸) and that it has an influence on fragment binding in fragment-based drug discovery (Skaist Mehlman *et al.*, 2023 ▸; Huang *et al.*, 2022 ▸). This should come as no surprise, since 100 K is very far away from the temperature of 310 K to which most human proteins are adapted. Nonetheless, structure-based drug discovery is almost exclusively carried out under cryogenic conditions, which begs the question of how reliable the results of such screens are with respect to biological reality (Skaist Mehlman *et al.*, 2023 ▸).

As mentioned above, SX, either at XFELs or synchrotrons, offers the possibility of collecting data at room temperature without the detrimental effects of rotational crystallography under noncryogenic conditions. In order to overcome cryo-bias in structure-based drug discovery, the logical step would be to use SX for this purpose. Initially, long data-collection times per data set and massive sample consumption prevented the high-throughput approaches needed for large drug and fragment screenings. However, it has been shown that high-quality data sets can be recorded in less than 3 min at a synchrotron, requiring only 3 µl of protein crystal suspension per data set (Zielinski *et al.*, 2022 ▸). Under these conditions, a standard fragment screen, consisting of 96 compounds, such as F2X-Entry (Wollenhaupt *et al.*, 2020 ▸), could in principle be collected within about 5 h, requiring only about 300 µl of microcrystalline slurry. Using a faster detector and collecting at 133 Hz instead of 25 Hz, this could be carried out in just one hour, using about 60 µl of sample.

Recently, it was shown that SSX at room temperature can be used to accurately identify fragments and larger ligands for a pharmaceutically relevant target protein (Dunge *et al.*, 2023 ▸). For this, a fixed-target approach was used for sample delivery to the X-ray beam focus on the BioMAX beamline at MAX IV, Lund, Sweden. Rather than screening a standard fragment library, the focus here was on a small selection of compounds and a comparison with results from data collection under standard cryogenic conditions. This comparison revealed mostly similar binding poses, with a notable temperature-dependent difference in the binding pose of one fragment. Moreover, differences in the number of water molecules that could be modeled and isotropic atomic displacement parameters could be observed (Dunge *et al.*, 2023 ▸).

SX is not limited to measurements at room temperature: it can in principle be conducted at any temperature that is deemed to be useful for the system under investigation, for example at 310 K for human proteins or for proteins from pathogens active in and against humans, or at even higher temperatures for proteins from thermophiles. It could also be interesting to work at lower temperatures to slow down enzymatic reactions and to make them observable for mix-and-diffuse methods.

Recent years have seen an increase in data collection above room temperature using rotational crystallography. Before 2020, only 12 structures from data collected above 300 K had been deposited in the PDB, and in the last four years 28 structures were added (or 18 to 46 if structures that seemed to be the result of a ‘warm’ experimental station at SACLA are included). These experiments yielded interesting insights into protein structure and dynamics (de Sá Ribeiro & Lima, 2023 ▸; Otten *et al.*, 2020 ▸; Ebrahim *et al.*, 2022 ▸) and altered ligand-binding properties (Huang *et al.*, 2022 ▸; Schuller *et al.*, 2021 ▸). Using a specifically designed enclosure to be able to control parameters such as humidity and temperature precisely, SX experiments at elevated temperatures ranging from 303 to 353 K could be conducted for the first time (Mehrabi *et al.*, 2021 ▸). This was combined with LAMA (liquid application method for time-resolved analysis) mix-and-diffuse (Mehrabi, Schulz, Agthe *et al.*, 2019 ▸) data collection for combined temperature- and time-resolved 5D-SSX of the catalytic reaction of xylose isomerase after addition of the substrate glucose (Mehrabi *et al.*, 2021 ▸).

## 3. Challenges

There are severe challenges which currently prevent the more widespread use of SSX, SFX and their combination with time-resolved data collection to unravel the structure and dynamics of biological macromolecules. In this section these challenges are discussed, as well as prospects for overcoming all or some of these challenges.

### 3.1. Sample preparation, delivery and consumption

Perhaps the greatest obstacle to successful SX experiments today, despite all of the efforts towards improvement during the past 15 years, is related to all aspects surrounding sample handling. In traditional crystallography all of these aspects have been resolved in the past 50 years and the last big change was the deployment of robots for crystal mounting at beamlines and automated centring of crystals. Nowadays, the whole process from purifying protein to structure solution is semi-automated. Nobody needs to spend long nights at a synchrotron beamline to collect data any more, and expertise in the fundamentals of crystallography is no longer a requirement for obtaining structures. Less than a microlitre of protein solution is required per crystal, there are standardized methods for crystallization and harvesting, and the collection of a full data set just takes a minute: from this perspective, SX seems to be comparatively crude and underdeveloped. To encourage more scientists to use the method, SX should clearly be adapted to serve the needs of scientists. This means that methods for producing highly concentrated, homogenous suspensions of microcrystals should be optimized and standardized. It should be as easy to obtain crystals for SSX or SFX as it is to obtain a larger crystal for rotation crystallo­graphy. There are now initial approaches which point in the right direction (Dunge *et al.*, 2023 ▸; Beale *et al.*, 2019 ▸; Stubbs *et al.*, 2024 ▸). A next step could be the introduction of commercially available kits developed for the production of high-quality microcrystals for SX.

**Figure 3:**
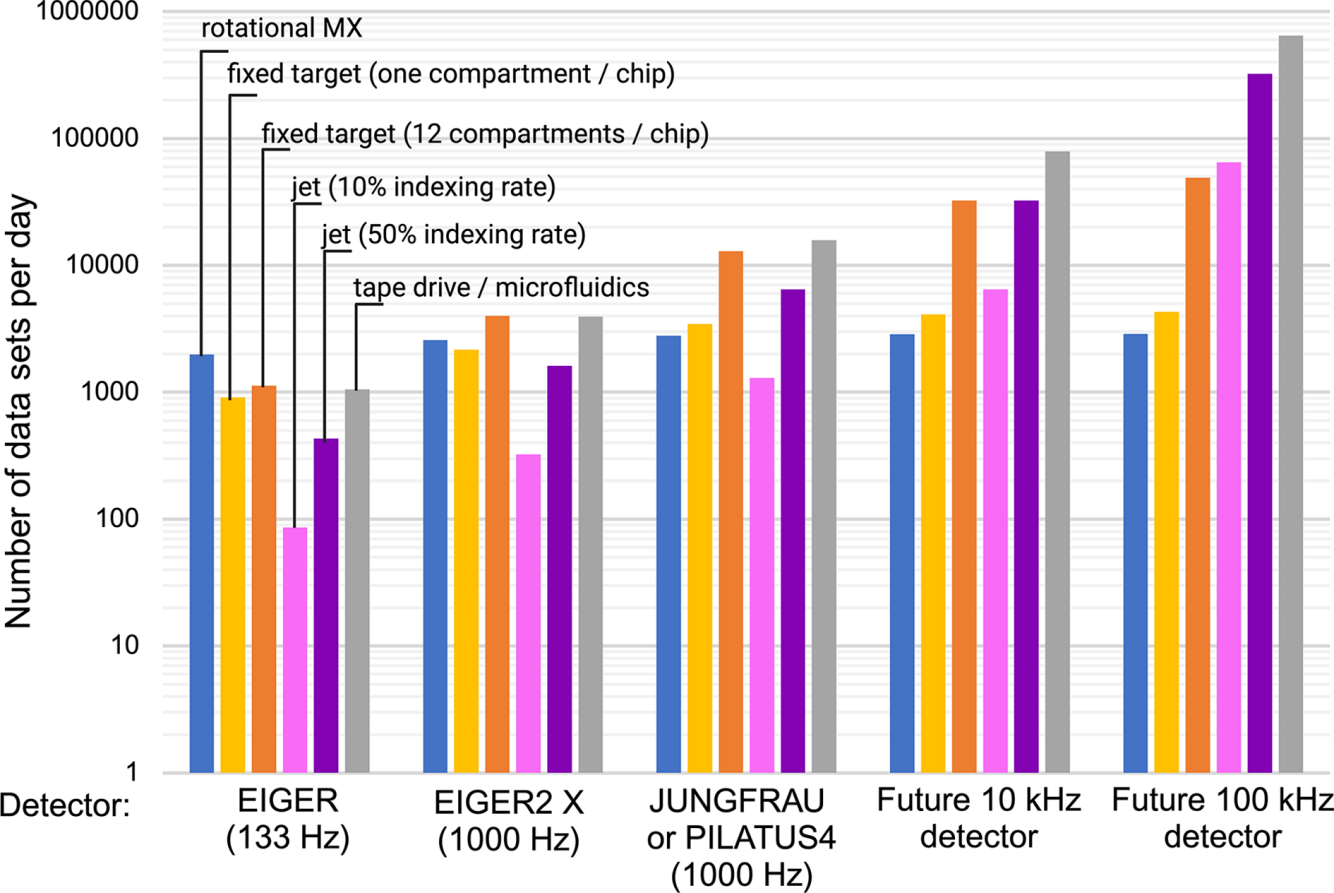
High-throughput compatibility of various sample-delivery methods for SSX/SFX compared with rotational macromolecular crystallography (MX) and how these scale with detector developments towards higher frame rates. The assumptions made were that 10 000 indexed frames are required for an SSX/SFX data set and that 1800 images need to be recorded for a data set from rotational MX. Furthermore, based on the literature (Bosman *et al.*, 2024 ▸; Zielinski *et al.*, 2022 ▸), we assumed a 100% indexing rate (indexable images per recorded images) for fixed-target sample delivery and tape drive/microfluidics. For liquid jets indexing rates of greater than 80% have been reported (Williamson *et al.*, 2023 ▸), but showing two cases, based on either a 10% or 50% indexing rate, seemed to be more realistic for most experiments. For rotational MX and fixed targets, we assumed the time it takes a robotic arm to exchange the sample to be 20 s. In the case of rotational MX we factored in another 10 s for crystal centring for each data set. For the tape drive/microfluidics we assumed 2 h downtime per day for the exchange of sample/tape/microfluidics. For all jets and tape drive/microfluidics in the 100 kHz detector case, we assumed 6 h of downtime per day. The number of data sets per day is plotted on a logarithmic scale. As can be seen, multi-compartment fixed targets and high-indexing jets are excellent in terms of high-throughput capability up to a 10 kHz frame rate. Single-compartment fixed-target chips and rotational MX are already not now competitive when detectors that are already available, such as JUNGFRAU or PILATUS4, are being used. Note that for SSX/SFX experiments further developments are required in order to unlock the full potential in terms of high-throughput capability. (Created with BioRender.com.)

Improving the attractiveness of SFX/SSX also means providing easy access to, and lowering barriers for, sample delivery. Fixed-target sample delivery for SX (Roedig *et al.*, 2017 ▸; Bosman *et al.*, 2024 ▸) is conceptionally very close to mounting a crystal in a loop for rotational crystallography, especially in the more recent foil-on-foil (or sheet-on-sheet) modifications (Doak *et al.*, 2018 ▸). Here, a first step to even more user-friendliness is the community-developed ‘standard descriptor for fixed-target serial crystallography’ (Owen *et al.*, 2023 ▸). A next step could be the adaptation of automatic sample exchange for fixed-target chips. Sample holders can be pre-loaded, stored under conditions preventing disintegration of the crystals and loaded one after another by a robotic arm. This would enable high-throughput data collection comparable with today’s rotational crystallography. Initial steps in this direction have been taken at the SwissMX endstation at SwissFEL (SwissFEL, 2024 ▸) and at HiPhaX at PETRA III/DESY (DESY, 2024 ▸). However, at some point the need for the exchange of fixed-target chips will be rate-limiting for both SSX and SFX, given the introduction of faster detectors that are currently under development (Fig. 3 ▸). Sample consumption using fixed-target supports for SFX or SSX is a little higher than that for rotation crystallography, with 3–100 µl of crystalline slurry usually being required per chip (Roedig *et al.*, 2015 ▸; Doak *et al.*, 2018 ▸; Schulz *et al.*, 2018 ▸), compared with the 400 nl to 4 µl used for typical crystallization droplets to grow larger crystals for rotation crystallography.

For many time-resolved experiments, however, the use of a high-viscosity extruder is the sample-delivery method of choice. Viscous sample delivery offers a sample efficiency similar to fixed-target sample delivery (Carrillo *et al.*, 2023 ▸), although the use of such extruders still requires experts, despite all of the efforts to improve them for better handling (Wranik, Kepa *et al.*, 2023 ▸). The same is true for liquid jets (Oberthuer *et al.*, 2017 ▸; Knoška *et al.*, 2020 ▸), where high sample consumption (50 µl to several millilitres per data set) additionally complicates matters, as well as challenges with liquid handling. Detailed investigations into fluid dynamics (Zahoor *et al.*, 2024 ▸), further design improvements and methods for the rapid exchange of both nozzles and samples are required to overcome these challenges, also necessitating dedicated beamtimes at XFELs for methods development.

Conveyor-belt-based hybrid methods (Butryn *et al.*, 2021 ▸; Zielinski *et al.*, 2022 ▸) or microfluidic sample-delivery systems (Ghosh *et al.*, 2023 ▸) are compatible with future high-throughputapproaches (Fig. 3 ▸) and time-resolved measurements, consume relatively small amounts of sample (comparable to fixed-target chips) and are less prone to clogging than liquid jets. In a systematic investigation of sample consumption, data-collection time and data quality, it was shown that a data set could be collected in 160 s, requiring less than 3 µl of sample (Zielinski *et al.*, 2022 ▸). It would be worth developing these hybrid sample-delivery methods further for easy and low-level access to users from the structural biology community.

### 3.2. Software and data analysis

Software for the initial stages of SX data processing such as hit finding, indexing, integration, scaling and merging has seen much development in the past years, with two main packages emerging for this purpose, *CrystFEL* (White *et al.*, 2016 ▸) and *cctbx.xfel*/*DIALS* (Hattne *et al.*, 2014 ▸), both of which are under constant development. Expert knowledge is no longer required for these stages of data processing. Improvements in software, both at the indexing and at the merging stage, have made SX more efficient: in many cases only 1000–10 000 indexed images need to be integrated and merged to obtain high-quality data sets (Zielinski *et al.*, 2022 ▸; Mehrabi, Schulz, Dsouza *et al.*, 2019 ▸). A novel approach to merging, based on a Bayesian model, has recently been introduced (Dalton *et al.*, 2022 ▸) and can take indexing/integration results, amongst others, from *CrystFEL* and *DIALS* as input.

However, refinement of the geometry of the experiment still remains very challenging (Yefanov *et al.*, 2015 ▸; Brewster *et al.*, 2018 ▸), including refinement of the beam center, of the sample-to-detector distance and of the detector geometry. Compared with rotational crystallography, this is complicated by the fact that each crystal is in a random orientation and thus there is no relation between subsequent diffraction images. In addition, most detectors used in SFX consist of multiple panels, which require not only accurate centring but also identification of the relative positions between the panels. The level of difficulty increases further if the detector used is an integrating detector with built-in gain switching. In addition to this, many detectors require initial image correction for the correct interpretation of measured intensities. Thus, today it is still not trivial to go from recorded diffraction events to merged intensities for downstream crystallographic refinement and model building.

Model building and crystallographic refinement are usually carried out with the same robust tools as in rotational crystallography. Challenges arise from the fact that this downstream software was mostly written for the purpose of structure determination of static structures, with one data set yielding one unambiguous structural model. This is not true for most time-resolved data sets, just as it is not true for most crystallographic data sets, especially when collecting data under noncryogenic conditions. Here, it is still rather challenging to model conformational heterogeneity, especially if heterogeneity means that not just some side chains but even entire loops exist in multiple conformations. Programs to aid with this have been developed in the past 15 years, such as *FLEXR* (Stachowski & Fischer, 2023 ▸), *qFit* (Wankowicz *et al.*, 2023 ▸) and ensemble refinement (Ploscariu *et al.*, 2021 ▸). Here, further utilization of molecular-dynamics simulations (Klyshko *et al.*, 2024 ▸) and an implementation of the paradigm of conformational heterogeneity into standard refinement pipelines could be the next step. Another special case is the identification of weak binders, where electron density identifying the binders is often only visible as the difference from an average of many data sets without the binder (Weiss *et al.*, 2022 ▸).

For time-resolved data sets containing mixed states it is often not possible to place one unambiguous structural model into the electron density. Instead, in the case of isomorphous data sets (Rould & Carter, 2003 ▸), initially weighted isomorphous difference electron-density (DED) maps are calculated (|*F*_o,light_ − *F*_o,dark_|, φ_calc,dark_), the occupancy of the triggered state is estimated and extrapolated structure-factor amplitudes (ESFAs) can be calculated (De Zitter *et al.*, 2022 ▸). Refinement is then carried out against these ESFAs, mimicking the data that would be obtained from 100% occupancy of the triggered state. This is widely used; however, the resulting maps and model-quality metrics are inferior compared with standard crystallographic refinement (Genick, 2007 ▸). There are cases in which the native and activated data sets are non-isomorphous, for example due to unit-cell changes upon the binding of a ligand. In this case neither isomorphous |*F*_o,light_ − *F*_o,dark_|, φ_calc,dark_ DED maps nor ESFAs can be calculated. Instead, *mF*_o_ − *DF*_c_ omit difference maps have to be used for initial assessment and modeling of the activated state, followed by refinement (Olmos *et al.*, 2018 ▸), which can be tricky or even impossible in the case of low occupancy of the activated state or multiple activated states at low occupancy. An alternative to detect structural differences between a ground-state and a perturbed-state data set could be *MatchMaker* (Brookner & Hekstra, 2024 ▸). Here, DED maps can be calculated irrespective of the isomorphism of the data sets. It first improves the phases of the activated-state data set through rigid-body refinement of the ground-state starting model against the activated-state structure-factor amplitudes, followed by the calculation of electron-density maps for the activated-state and ground-state data, alignment of these two maps and finally computation of a DED map. In the case of high-resolution, high-quality data sets at high occupancy of the activated state, iterative multicopy refinement at various occupancies of the activated state might be an alternative to avoid the use of ESFAs (Barends *et al.*, 2024 ▸).

### 3.3. Reaction initiation

As mentioned above, there is a disparity between the readiness of SSX for mix-and-diffuse time-resolved experiments and the lack of published results. There should be a sufficient number of enzymes for which the catalytic cycle proceeds in the regime that can be observed using this method (a few milliseconds to seconds). It could be that the analysis of results is slow due to problems with software and data interpretation, thus delaying publication. However, despite all of the developments (Mehrabi, Schulz, Agthe *et al.*, 2019 ▸; Calvey *et al.*, 2019 ▸; Knoška *et al.*, 2020 ▸; Vakili *et al.*, 2022 ▸), reaction initiation is not really trivial for chemical triggering. Challenges to overcome are the mixing process as such, the susceptibility of the crystals to diffusion of the trigger (Pletzer-Zelgert *et al.*, 2023 ▸; Schmidt, 2013 ▸) and, as a combination of both, uniformity of the trigger. If the trigger is non-uniform a mixture of states will be present, from which it is very difficult to discern something meaningful. Using smaller crystals, as well as investigations into the bottlenecks of diffusion, will help here. Submicron-sized protein crystals can already be used at XFELs and will be usable at diffraction-limited sources (such as ESRF-EBS or PETRA IV in the future) if beamline design makes full use of the possibility of focusing the X-ray beam to a submicrometre spot size at full intensity.

As can be seen by the plethora of published studies using light activation for time-resolved measurements, the problem of uniform reaction initiation does not exist with modern lasers, which can be controlled very precisely. Caution has to be exercised regarding pre-exposure to light, which can occur with any sample-delivery method. In a recent study, it was found that one of the ‘dark’ states collected was indeed a light-exposed state. The authors used a light–dark1–dark2 data-collection pattern and additionally a data set collected without laser activation as a reference to resolve this (Chretien *et al.*, 2024 ▸). However, light activation bears another, severe, problem, which is related to the response of the protein under investigation to the laser pulse. Depending on the laser settings, the protein target and the crystal size, only a small fraction of the protein molecules in the crystal might be excited by the pump laser. Consequently, the crystallographic occupancy of the excited state is too small and analysis of the time-dependent evolution of electron density fails. How large the excited fraction needs to be depends strongly on the data quality, meaning that higher resolution and a better signal-to-noise ratio are critical in this regard. Optimizing crystallization to obtain small, uniform and high-quality crystals is one prerequisite for a successful experiment. In addition, in general a higher quality data set can be acquired in SFX by collecting more data and by careful optimization of the geometry of the experiment (see above). To increase the fraction of protein molecules in the crystal being excited to a productive state (the state which allows the protein to enter the reaction pathway under investigation), careful optimization of the laser settings (laser wavelength, peak power and pulse length) is required. For this, it might be helpful to conduct transient absorption spectroscopy before and in addition to trSFX experiments (Do *et al.*, 2024 ▸; Christou *et al.*, 2023 ▸). To allow maximum occupancy of the excited state, the size of the crystals used should be no larger than the penetration depth of the pump laser (Grünbein *et al.*, 2020 ▸) in the crystalline material. Despite all efforts to optimize the experiment, it can be necessary to use laser settings at which the laser power should, theoretically, result in nonlinear processes and possible excitation of off-pathway states. As shown in a recent review (Brändén & Neutze, 2021 ▸), many successful trSFX experiment have been conducted in this regime. This practice has been criticized for quite some time, because the structural differences between the resting and activated state observed at high fluence density might be real, yet might also be the result of two-photon effects and thus not representative of the biological truth (Grünbein *et al.*, 2020 ▸; Miller *et al.*, 2020 ▸). A further systematic investigation into such effects has now been published (Barends *et al.*, 2024 ▸) and there is an ongoing debate in the field, as highlighted by a discussion (Neutze & Miller, 2024 ▸) on the implications of this systematic investigation.

## 4. Outlook

**Figure 4:**
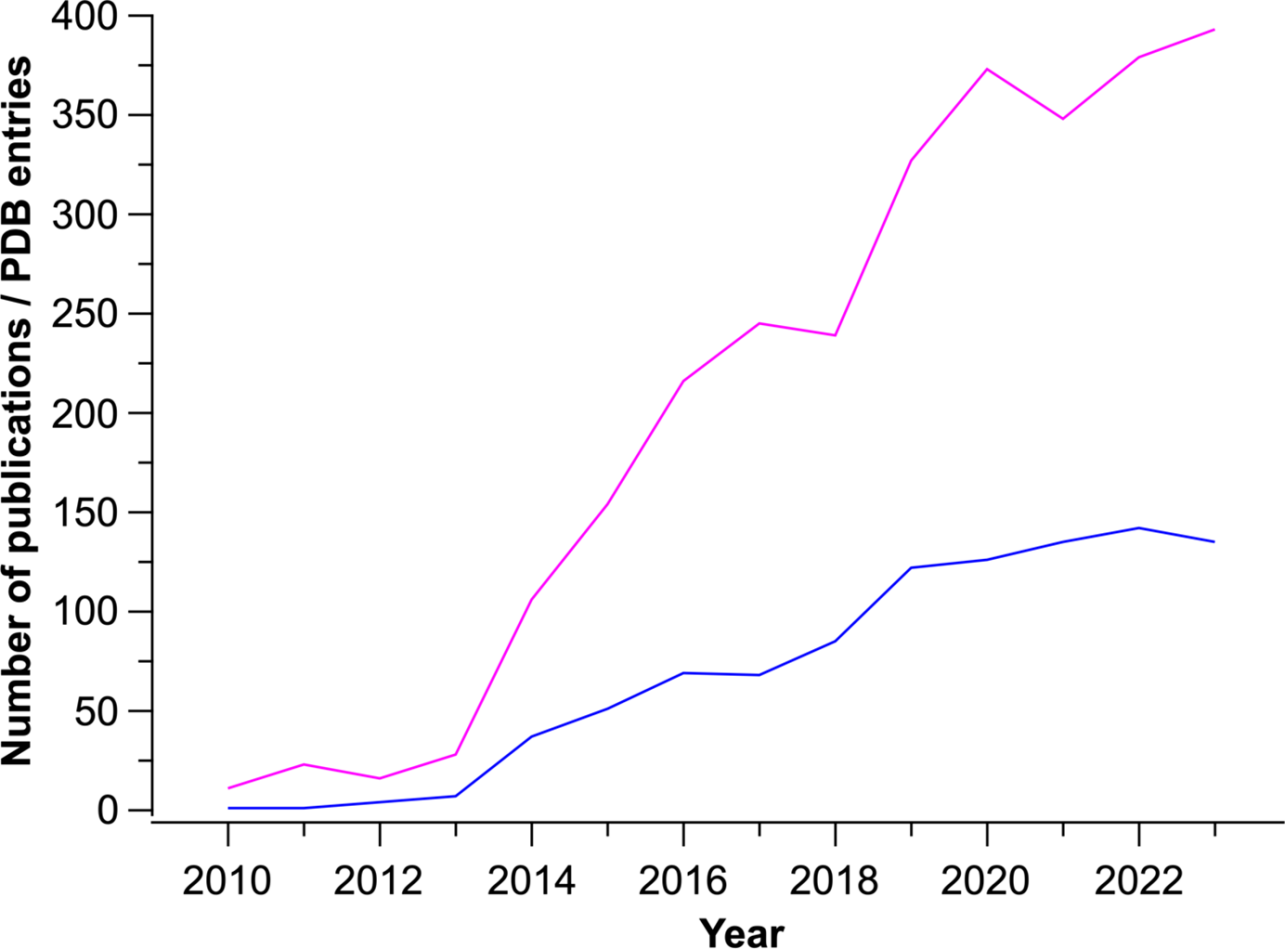
Development of the number of new PDB entries per year from SFX/SSX experiments (blue line) and the number of publications mentioning ‘serial crystallography’ since 2010 (pink line). The number of publications is based on a query using Google Scholar. For the number of PDB entries the deposition date, rather than the release date, was used.

Tr-SX has made tremendous progress within the last few years, as can be seen from the increasing number of published results (Fig. 4 ▸). At the same time, it is still a small research community and access to the tools is not as easy as it could and should be. The availability of protein crystal screening (PCS) beamtimes at many XFEL facilities is a great step forwards in this direction. This is particularly true if the PCS beamtimes are organized in a way such that scientists can just bring their sample and do not need to worry about sample delivery and data processing. The wider availability of SSX endstations will further lower barriers in future. Facilities operating XFELs and synchrotrons are urged to consider different schemes of access, offering for example the possibility of joint SFX and SSX proposals and more flexibility in beamtime schedules. SFX and SSX should become standard tools for structural biology, just as rotational crystallography, cryoEM or mass spectrometry are today. For this, some or all of the challenges above must be overcome. SX data collection in the future should be as easy or easier to conduct than cryo-crystallo­graphy, by implementing robust data-collection pipelines, including robotics for automated sample exchange and autosamplers for high-throughput microfluidic sample delivery. Furthermore, additional dedicated endstations for SSX should be established at synchrotrons and existing beamlines should be retrofitted to allow easy access to SSX. SX should deliver as good or better data quality (resolution, interpretability and representation of biological truth), should be as automated, as fast or faster, and require the same amount of sample or less. For this, software needs to be developed further so that it can process data on-the-fly, and automated refinement pipelines should also be established that can cope with the expected high output from such experiments. The data need to be easily accessible and presented in a way that non-experts in SX can interpret the results. If all of this can be achieved, SX will prevail, because only SX can make full use of today’s radiation sources and tomorrow’s detectors and at the same time realize its enormous potential in unraveling the structural dynamics of biological macromolecules.
